# Near-Infrared Fluorescence Imaging of EGFR-Overexpressing Tumors in the Mouse Xenograft Model Using scFv-IRDye800CW and Cetuximab-IRDye800CW

**DOI:** 10.1155/2022/9589820

**Published:** 2022-04-14

**Authors:** Abolfazl Amini, Yaghoub Safdari, Fatemeh Tash Shamsabadi

**Affiliations:** ^1^Department of Medical Biotechnology, Faculty of Advanced Technologies in Medicine, Golestan University of Medical Sciences, Gorgan, Iran; ^2^Medical Cellular and Molecular Research Center, Golestan University of Medical Sciences, Gorgan, Iran; ^3^Golestan Research Center of Gastroenterology and Hepatology, Golestan University of Medical Sciences, Gorgan, Iran

## Abstract

EGFR (epidermal growth factor receptor) is overexpressed in a variety of human cancers (including squamous cell carcinoma of head and neck, colon cancer, and some breast cancers) and therefore is regarded as an ideal target for cancer therapy or imaging purposes. In the current study, we produced a scFv-based near-infrared probe (called cet.Hum.scFv-IRDye-800CW) and evaluated its ability in recognizing and imaging of EGFR-overexpressing tumors in a mouse model. Like the molecular probe consisting of its parental antibody (cetuximab, an FDA-approved monoclonal antibody) and IRD800CW, cet.Hum.scFv-IRDye-800CW was able to recognize EGFR-overexpressing tumors in mice. cet.Hum.scFv-IRDye-800CW was found to be superior to the cetuximab-based probe in imaging of mouse tumors. The tumor-to-background ratio and blood clearance rate were higher when cet.Hum.scFv-IRDye-800CW was used as an imaging probe.

## 1. Introduction

Surgical resection of the primary solid tumors in patients plays an important role in cancer therapy. Positive margins remaining after an incomplete resection significantly contribute to the disease recurrence [1, 2]. Traditional tumor imaging techniques such as magnetic resonance imaging, computed tomography, and X-ray are not sensitive and specific enough to differentiate tumor tissues from surrounding normal tissues intraoperatively [3]. Fluorescence image-guided surgery, as a promising technique for intraoperative monitoring, provides real-time guidance for surgeons during surgery [4]. Fluorescent targeting agents, such as fluorescent dye-conjugated monoclonal antibodies, make it possible to image tumors with high specificity [5, 6].

Many studies have highlighted the clinical benefits of fluorescence-guided surgery of patients with solid tumors, where molecular probes made from antibody molecules and a fluorescent dye are used [4, 7–16]. For instance, molecular probe consisting of anti-EGFR monoclonal antibodies and fluorescent dyes has been shown to be a good probe to detect human head and neck tumors [4, 17]. The epidermal growth factor receptor (EGFR) is a transmembrane cell surface protein belonging to HER family tyrosine kinase receptors that plays a pivotal role in proliferation, migration, survival, and invasion of cancer cells [18–20]. EGFR is frequently overexpressed in various cancers including head and neck [21], non-small-cell lung [22], breast [23], and cervical and colorectal cancers [24, 25], so it can be regarded as an ideal molecular target for use in cancer imaging and therapy.

Currently, there are four major EGFR-targeting monoclonal antibodies, namely, panitumumab, nimotuzumab, cetuximab, and necitumumab. Panitumumab, nimotuzumab, and cetuximab have been conjugated to various tracers, and the resultant conjugates are currently under clinical trials [26]. For instance, Rosenthal and colleagues have evaluated the capacity of the cetuximab-IRDye800CW conjugate in imaging of head and neck cancer [4, 17].

Tumor-detecting agents are of great interest for surgical oncologists. OTL-38 is an FDA-approved agent that is used for detection of ovarian cancer during fluorescence-guided surgery [27]. Antibody-fluorescent dye conjugates are also of great interest for tumor detection. Full antibody or antibody fragments can be used for this purpose. Despite having several advantages (e.g., high affinity and specificity toward their pertinent antigens), full-length antibodies are relatively large molecules (ranging 140-150 kDa). This drawback may limit their application when dealing with solid tumors [28, 29]. Long half-life and limited penetration of full-length antibodies can lead to high background levels, resulting in a low tumor-to-background ratio [3, 30]. Hence, smaller antibody molecules will be of higher interest for cancer imaging. Among anti-EGFR monoclonal antibodies, cetuximab (in conjugation with IRDye800CW) has been evaluated in both animal and clinical trial studies and shown to be an effective agent for EGFR-bearing tumors [17, 21], so we used it as a control in this study. Single-chain antibodies, summarily called scFvs, are one of the smallest antibody fragments with satisfied affinity toward antigens. By having only two variable regions, scFv molecules are regarded as small antibody molecules with molecular weight of ≤30 kDa [29]. Because of their relatively smaller size, lower immunogenicity, and easy production, scFvs have attracted enormous attention for several applications [29, 31]. Moreover, due to the absence of the Fc fragment in scFvs, the off-target effects are decreased on Fc-receptor-positive cells. Gong and colleagues reported that IRDye800CW-conjugated anti-TAG-72 scFv provides rapid and specific recognition of colorectal tumors in the mouse model [32].

In our previous work, we produced and evaluated the binding capacity of a germline-humanized recombinant anti-EGFR scFv [33]. In the current study, we have evaluated the ability of this scFv in recognizing EGFR-overexpressing tumors in the mouse model when conjugated to IRDye800CW. IRDye800CW is cleared from bloodstream by kidneys [34]. IRDye800CW is fluorescent dye with absorption and emission wavelengths in near-infrared spectrum [35]. Medical optical imaging plays an important role in biomedicine and surgery. However, due to severe light scattering and tissue autofluorescence in the visible light spectrum (650-900 nm), conventional fluorescent dyes (e.g., FITC) often have a poor signal-to-noise ratio (SNR) and low penetration ability and imaging sensitivity. Near-infrared spectrum (700–900 nm) has been reported to have more tissue penetration ability and show higher SNR. Low scattering rate and minimal tissue-based autofluorescence of near-infrared dyes make it possible to obtain better images of tumors [36, 37]. In the current study, we compare the ability of cetuximab and an anti-EGFR scFv in imaging of EGFR-overexpressing tumors after conjugation to IRDye800CW near-infrared dye.

## 2. Materials and Methods

### 2.1. Reagents

Cetuximab (concentration of 5 mg/mL, total volume of 20 mL) was prepared from Red Cross Pharmacy of Gorgan (Gorgan, Iran). IRDye800CW-NHS was purchased from LI-COR Biosciences, Lincoln (USA). Diaminobenzidine (DAB) tablet set (cat. no. T0440) and TMB (3,30,5,50-tetramethylbenzidine) (cat. no. D4293) were purchased from Sigma-Aldrich. Recombinant human EGFR protein (cat. no. ab155726) and anti-alpha tubulin antibody (cat. no. ab15246) were purchased from Abcam. HRP-Protein L (cat. no. M00098) and Ni-NTA Agarose (cat. no. 30210) were purchased from Qiagen (Germany) and GenScript Biotech (USA), respectively.

### 2.2. Cloning and Expression of cet.Hum.scFv

A humanized single-chain variable fragment (scFv) was the product of our previous study. We named the scFv as cet.Hum.scFv. The scFv-encoding sequence (consisting of heavy-chain variable domain- (VH-) encoding sequence, linker-encoding sequence, and light chain variable domain- (VL-) encoding sequence) has been inserted in the cloning region of pET22b(+) bacterial expression vector between NcoI and XhoI restriction sites. Expression of the sequence results in the production of a 27 kDa scFv in VH-linker-VL format [33].

### 2.3. Expression and Purification of cet.Hum.scFv

For recombinant protein expression, recombinant pET22b(+)-cet.Hum.scFv vector was transformed to *Escherichia coli* (*E. coli*) BL21 (DE3) cells using the heat shock method. *E. coli* cells were cultured in a conical flask containing 50 mL Luria-Bertani (LB) medium supplemented with 100 *μ*g/mL ampicillin and allowed to grow for 3 h at 37°C. When OD_600_ reached to 0.5, IPTG (isopropyl-*β*-d-1-thiogalactoside) was added to the flask (final concentration of 0.1 mM) to induce recombinant cet.Hum.scFv expression. After an overnight growth at 18°C, the medium was centrifuged at 4000 *g* for 15min and the resultant bacterial pellet was suspended in lysis buffer (containing 50 mM Tris-HCl pH 7.5, 200 mM NaCl, 1 mM PMSF) for sonication. Sonication was carried on ice for 30 cycles with 30 s intervals at amplitude 80. cet.Hum.scFv was purified using Ni-NTA agarose according to the company's (Qiagen) protocol and stored at −20°C for subsequent use. The Bradford assay was used to quantify cet.Hum.scFv concentration in the solution.

### 2.4. Cell Lines and Cell Cultures

A-431 and U-87 MG (U-87) cell lines were obtained from the Cell Bank of Pasteur Institute (Tehran, Iran). A-431 is a human skin cancer cell line overexpressing EGFR [38]. U-87 is a brain cancer cell line with low EGFR expression level [39]. Both cell lines were cultured at 37°C with 5% CO_2_ in Dulbecco's modified Eagle's medium (DMEM; Gibco) supplemented with 10% fetal bovine serum (FBS), 1% penicillin/streptomycin (pen/strep), and 25 *μ*g/mL gentamicin. A-431 and U-87 MG were used as high and low EGFR-expressing cell lines, respectively.

### 2.5. Western Blotting

The cells were lysed by sonication (5 s) while being suspended in modified lysis buffer (50 mM Tris, 1 mM EDTA, 150 mM NaCl, 0.1% SDS, 1% Triton X-100, and 100 mM PMSF, pH = 7.5) [40, 41]. Cellular debris was removed by centrifugation at 10000 g for 10 min, and the supernatant was stored at -80°C after being aliquoted.

A-431 and U-87 cell lysates were run on an SDS-PAGE, and separated proteins were transferred to PVDF membranes. To prevent unspecific reactions, the membranes were immersed in blocking buffer (TBS buffer containing 3% bovine serum albumin (BSA) and 0.05% Tween-20). After an overnight incubation at 4°C, the membranes were washed with TBS buffer and reacted with cet.Hum.scFv (50 *μ*g/mL), cetuximab (30 *μ*g/mL), or anti-alpha tubulin (20 *μ*g/mL) for 2 hours at room temperature (RT). After another round of washing with TBS, the membranes (those reacted with cet.Hum.scFv or cetuximab in the previous stage) were reacted with HRP-Protein L solution (final concentration of 0.5 *μ*g/mL) for 1 hour at RT. Finally, the membranes were reacted with freshly prepared DAB solution to visualize the spots.

### 2.6. IRDye800CW Antibody Labeling

cet.Hum.scFv and cetuximab were labeled with IRDye800CW (IRdye800CW-NHS ester, LI-COR Biosciences) according to the manufacturer's guidelines [42, 43]. Briefly, both antibodies were incubated with IRDye800CW (antibody/dye molar ratio of 1/10) in potassium phosphate buffer (1 M, pH 8.5) while gently being shacked on a rotator at 4°C. After 2 h incubation, the solutions were dialyzed using dialysis bags (molecular weight cutoff 12-14 kDa) by overnight incubation at 4°C in the molar ratio in 1X PBS buffer (pH 7.0).

After dialysis, the dye-to-antibody ratio (degree of labeling (DOL)) was measured using a Picodrop Microliter UV/Vis spectrophotometer at the absorbance wavelengths of 280 nm and 774 nm (*A*_280_ and *A*_774_) [42].

### 2.7. Binding Assay (Antigen Saturation Assay)

Affinity of IRDye800CW-conjugated molecules was determined using ELISA. Briefly, 100 *μ*L of cell lysate (lysate of A-431 or U-87 MG cells) was added to each well on an ELISA plate for overnight incubation at 4°C. After three times washing with PBS, the wells were blocked by adding 250 *μ*L blocking buffer (PBS buffer supplemented with 3% BSA) for 2 h. After another rounds of washing with PBS, dye-conjugated molecules (cetuximab-IRDye800CW and cet.Hum.scFv-IRDye800CW) were added to the wells (100 *μ*L/well, final concentrations of 0.7 to 50 *μ*g/well) and allowed to react with the cell lysates for 2 h at 37°C. After three times washing with PBS (5 min each time), HRP-Protein L was added to each well (final concentration of 0.5 *μ*g/mL) and allowed to react with the content for 1 h. After washing the wells with PBS buffer as the previous stage, 100 *μ*L TMB was added to each well to obtain OD values of antibody-antigen interaction at 450 nm.

### 2.8. Animal Models and NIR Fluorescence Imaging

4-6-week-old female immunosuppressed BALB/c mice (prepared according to the protocol of Jivrajani and colleagues [44]) were used in this study. Mice were kept in standard cages under sterile housing conditions at 25°C, 60% relative humidity, and 12 h light/dark cycles, with food and water ad libitum. All animal experiments and anesthetic/euthanasia processes were performed in accordance with the institutional animal care and use committee (IACUC). Ethics approval for this study was obtained from the Golestan University of Medical Sciences (ethics registry number IR.GOUMS.REC.1398.001). For tumor induction, each immunosuppressed mouse (*n* = 48) received (as subcutaneous injection into the right hind flank region) 100 *μ*L FBS-free culture medium containing 8 × 10^6^ of either A-431 or U-87 cells. Tumor growth was monitored weekly using calipers until tumor size reached 10-15 mm. The mice were randomly divided into six different groups (6-8 mice per group): (1) A-431, PBS; (2) A-431, cet.Hum.scFv-IRDye800CW; (3) A-431, cetuximab-IRDye800CW; (4) U-87, PBS; (5) U-87, cet.Hum.scFv-IRDye800CW; and (6) U-87, cetuximab-IRDye800CW. The mice were then systemically injected through the tail vein with PBS, cet.Hum.scFv-IRDye-800CW, or cetuximab-IRDye-800CW (75 *μ*g in a total volume of 100 *μ*L). Mice were anesthetized with 2 mg ketamine and 0.2 mg xylazine injected into the peritoneal cavity and imaged at 0, 1, 4, 24, 48, 72, and 96 h after injection (hpi). NIR images were taken using the FluoVision optical imaging system (Tajhiz Afarinan Noori Parseh Co., Tehran, Iran) [45] equipped with a near-infrared specific filter set (part number IRDYE800-33LP-A-000, Semrock, USA) with excitation and emission wavelengths of 747 and 776 nm.

### 2.9. Statistical Analysis

The concentration of cet.Hum.scFv-IRDye800CW and cetuximab-IRDye800CW was calculated using the following formula: protein concentration (mg/mL) = [(A280 − (0.03 × A774))/*ε*_Protein_] × MW Protein × Dilution Factor. The degree of labeling was measured using the following formula: DOL = [A774/*ε*IRDye800CW] ÷ [(A280 − (0.03 × A774))/*ε*_Protein_]. The correction factor for the absorbance of IRDye800CW at 280 nm (equal to 3.0% of its absorbance at 774 nm) is 0.03. *ε*_Protein_ is the molar extinction coefficients for the protein. MW protein is the molecular weight of the protein. The dilution factor is the dilution of the labeled conjugate prior to measurement with a spectrophotometer. The molar extinction coefficient of IRDye800CW is 240,000 M^−1^ cm^−1^, and the molar extinction coefficients for the proteins (*ɛ*_Protein_) are 53,860 M^−1^ cm^−1^ (for scFv) and 217,440 M^−1^ cm^−1^ (for cetuximab). ELISA data was analyzed using GraphPad Prism 8 (GraphPad Software, San Diego, CA). The tumor-to-background ratio and fluorescent signal intensity were calculated using ImageJ (https://imagej.net/).

## 3. Results

### 3.1. Expression Purification of Human Single-Chain Fragment Antibody

**Figure 1:**
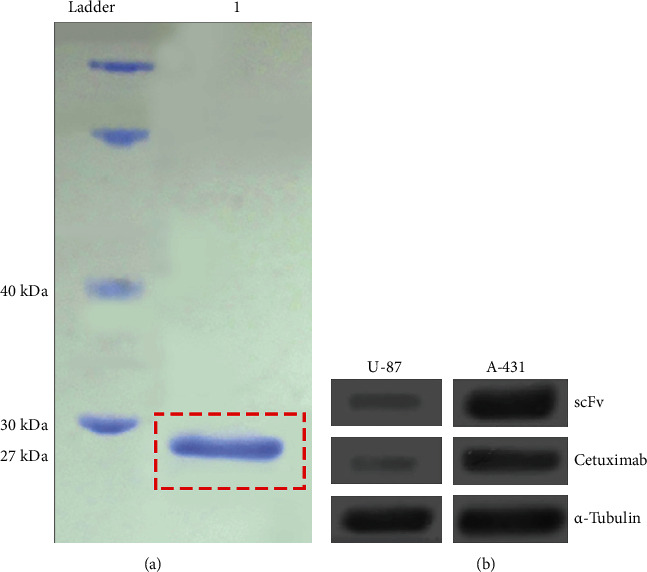
SDS-PAGE and western blotting results. (a) His-tag affinity chromatography purified cet.Hum.scFv. Protein bands of the same molecular weight (27 kDa) appeared in 1 lane. (b) The results of western blotting with the antibodies and A-431 and U-87 MG cells. Alpha-tubulin was used as the loading control. Both cet.Hum.scFv and cetuximab are able to form thick protein bands of approximately 175 kDa with A-431 cells, but not with U-87 MG cells.

**Figure 2:**
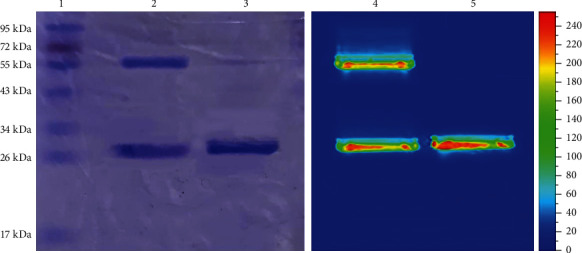
Characterization of IRDye800CW-labeled cet.Hum.scFv and cetuximab. SDS-PAGE image after Coomassie blue staining (lanes 1, 2, and 3 are protein ladder, cetuximab, and cet.Hum.scFv, respectively). The SDS-PAGE under near-infrared filter-equipped animal imaging system (lanes 4 and 5 are cetuximab and cet.Hum.scFv, respectively).

cet.Hum.scFv expression was carried out as described in our previous work and purified using NI-NTA resin (Figure 1(a)). As expected, both cet.Hum.scFv and cetuximab were found to detect EGFR molecules in the lysate of EGFR-overexpressing A431 cells lysates, but not significantly in the lysates of U-87 cells (Figure 1(b)). IRDye800CW NHS ester was conjugated to human cet.Hum.scFv and cetuximab (control antibody) by acylating the free primary amines, such as lysine residues in antibodies (Figure 2). The final protein concentration of cet.Hum.scFv and cetuximab after purification was 48.9 *μ*g/mL and 51.3 *μ*g/mL, and the DOL ratio was 1.983 and 2.128, respectively.

### 3.2. Cell-Binding Assay of cet.Hum.scFv, cet.Hum.scFv-IRDye, Cetuximab, and Cetuximab-IRDye

**Figure 3:**
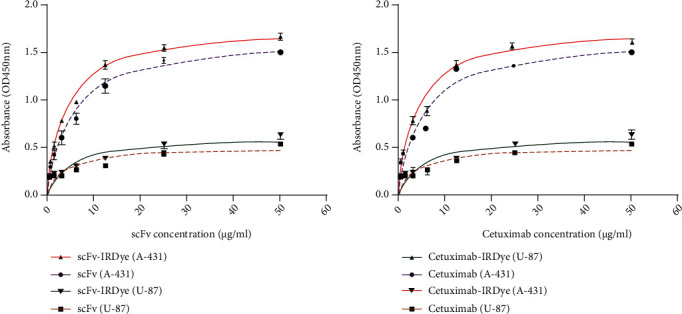
The saturation binding curves of IRDye800CW-labeled and unlabeled antibodies when reacting with A-431 and U-87 cells. All experiments were done in triplicate.

We measured the ability of labeled and unlabeled cet.Hum.scFv and cetuximab in recognizing A-431 and U-87 cells in ELISA (Figures 3(a) and 3(b)). For A-431 cells, *K*_d_ values of cet.Hum.scFv-IRDye800CW and cetuximab-IRDye800CW were calculated to be 21 nM (±0.5) and 24.3 nM (±0.9), respectively. Unlabeled cet.Hum.scFv and cetuximab had *K*_d_ values of 5.3 ± 1.3 and 4.9 ± 1.8 nM, respectively. No significant difference was found in *K*_d_ values between the cet.Hum.scFv and cetuximab (*p* > 0.05). No significant OD values were obtained when the antibodies reacted with U-87 MG cells (Figure 3).

### 3.3. In Vivo Tumor Imaging Using IRDye800CW-Conjugated Antibodies

**Figure 4:**
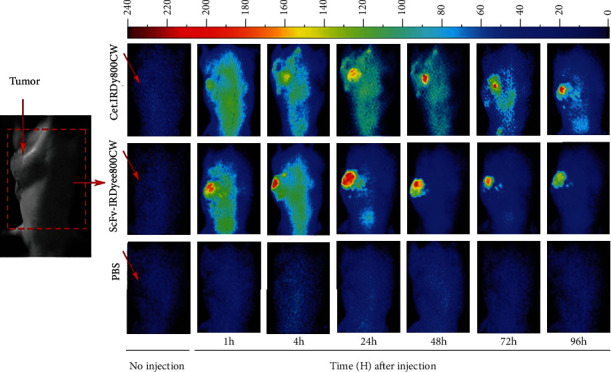
NIR fluorescence images of cet.Hum.scFv-IRDye800CW and cetuximab-IRDye800CW conjugates in mice bearing A-431 tumor xenografts. Images were obtained at noninjection and at 1, 4, 24, 48, 72, and 96 hours postinjection of 100 *μ*L for the cet.Hum.scFv-IRDye800CW and cetuximab-IRDye800CW (75 *μ*g). Scale bar changes are shown on the top. The degree of labeling or D/P ratio of cet.Hum.scFv-IRDye800CW and cetuximab-IRDye800CW was 1.983 and 2.128, respectively.

**Figure 5:**
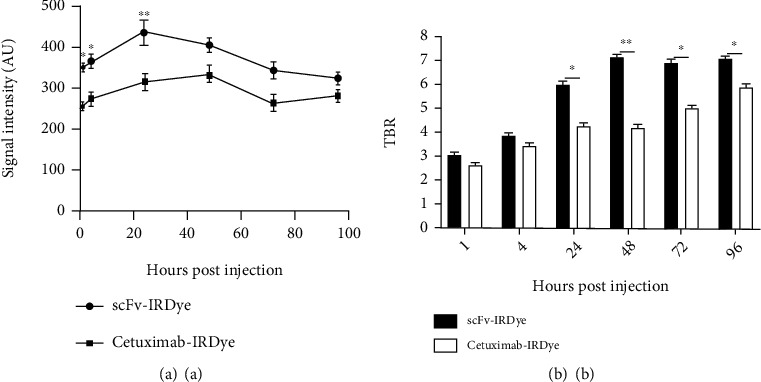
NIR fluorescence analysis of IRDye800CW-conjugated molecules after applying on A-431 tumor-bearing mice. (a) Intensity of fluorescent signals emitting from tumor tissues. (b) Tumor-to-background ratio analysis. Error bars represent mean ± standard deviation. The asterisks indicate significant differences between groups (^∗^*p* < 0.05, ^∗∗^*p* < 0.01).

Images taken after IRDye800CW-antibody injection at different times are shown in Figures 4 and 5. In the case of the mice bearing A-431 tumor xenografts, cet.Hum.scFv- IRDye800CW was found to produce more intense and condensed signal than cetuximab-IRDye800CW. cet.Hum.scFv-IRDye800CW continued to emit signal even at the fourth day of injection (96 h after injection). The maximum signal intensity occurred 24 h after injection (420 ± 40 au) (Figure 5(a)). cetuximab-IRDye800CW was also able to fluoresce inside the A-431 tumor xenografts. Like cet.Hum.scFv-IRDye800CW molecules, cetuximab-IRDye800CW continued to fluoresce even at the fourth day of injection. The maximum signal intensity for cetuximab-IRDye800CW occurred 48 h after injection (325 ± 34 au). Signal intensity for cet.Hum.scFv-IRDye800CW at 1, 4, and 24 h after injection was calculated to be 350 ± 10 au, 365 ± 20 au, and 435 ± 32 au, respectively. These values for cetuximab-IRDye800CW were calculated to be 254 ± 9.7 au, 272 ± 10 au, and 315 ± 22 au, respectively (see Figure 5(b) for statistical significance level). The tumor-to-background ratio was significantly higher in mice receiving cet.Hum.scFv-IRDye800CW than those receiving cetuximab-IRDye800CW. Pairwise comparison of these ratios for IRDye800CW-conjugated cet.Hum.scFv/cetuximab is shown as follows: 24 h after injection (5.9 versus 4.3), 48 h after injection (7.1 versus 4.2), 72 h after injection (6.9 versus 5), and 96 h after injection (7.1 versus 5.8) (see Figure 5(b)).

**Figure 6:**
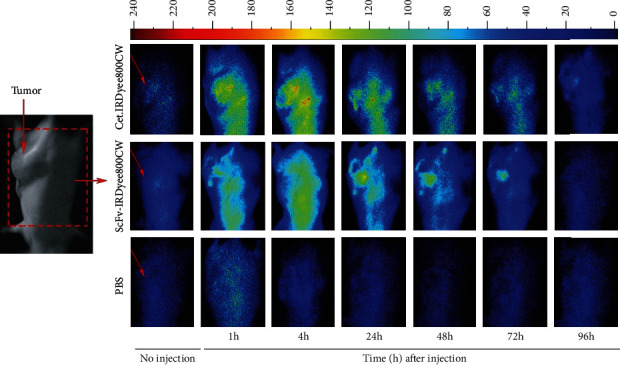
NIR fluorescence imaging of U-87 MG-bearing mice after injection of IRDye800CW-conjugated antibodies. Degree of labeling and amount of injection are the same as described in the Figure 4 caption.

**Figure 7:**
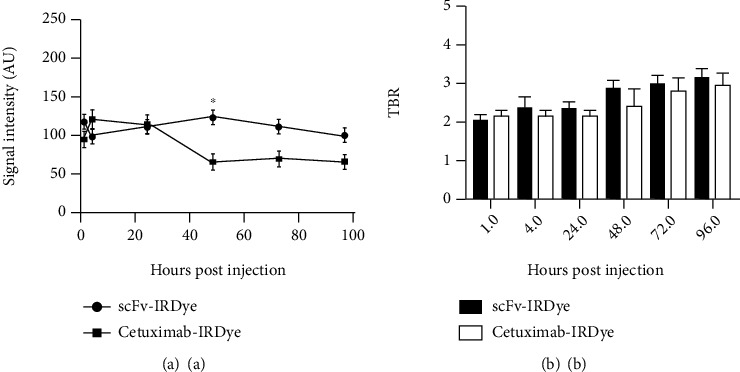
NIR fluorescence analysis of U-87 MG tumors receiving IRDye800CW-conjugated antibodies. (a) Signal intensity 24 h after injection; both antibodies raised the same signal intensity. The maximum difference in signal intensity occurred 48 h after injection; a significant difference at statistical level of 5% (^∗^*p* < 0.05). (b) Tumor-to-background ratio. There were no significant differences in the ratios of cet.Hum.scFv-IRDye800CW and cetuximab-IRDye800CW.

We studied the accumulation of the IRDye800CW-labeled humanized cet.Hum.scFv and cetuximab in U-87 MG tumor xenografts (Figure 6). cet.Hum.scFv-IRDye80CW-induced signal intensity in U-87 MG tumor xenografts (100-125 au) was significantly lower than that in EGFR-overexpressing A431 xenografts (278-420 au) (*p* ≤ 0.05) (Figure 7(a)). 1 h after injection, the tumor-to-background ratio in mice receiving IRDye800CW- cet.Hum.scFv was calculated to be 2, which raised to 3.2 after 72 h; the same trend was observed when U-87 MG-bearing mice received cetuximab-IRDye800CW; signal intensity (65-119 au) was lower than that emitting from A431 tumor xenografts (254 − 325 au) (*p* ≤ 0.05) (Figures 5(a) and 7(a)). The tumor-to-background ratio in mice receiving cetuximab-IRDye800CW did not exceed 3 (Figure 7(b)).

## 4. Discussion

In the current study, we compared the abilities of a humanized cet.Hum.scFv (approximately 27 kDa) and its full-length parental antibody (cetuximab, approximately 152 kDa) to see which one is better for tumor imaging when conjugated to IRDy800CW near-infrared fluorescent dye. The ability of IRDye800CW-conjugated cetuximab in detecting EGFR-overexpressing tumors has already been reported in both preclinical and clinical studies, and it has been found to be a suitable tumor-detecting agent [17, 21]. In the current study, we showed that cet.Hum.scFv-IRDye800CW can enter EGFR-overexpressing tumors in an efficient manner. The cet.Hum.scFv carries the same CDR loops of cetuximab, so it retains the antigen-binding ability, and due to its relatively smaller size, it is expected to be more prone to enter tumor tissues. Our results confirmed this hypothesis; we found that cet.Hum.scFv-IRDye800CW is more effective than its parent in entering tumor tissues and emitting stronger fluorescent signals. It takes longer time for larger proteins to disappear from blood stream following liver metabolism [46]. So, cet.Hum.scFv molecules should disappear in blood stream sooner than cetuximab, while still presenting in tumor tissues, where tumor cells with higher EGFR expression are present. This may be the reason why the tumor-to-background ratio differs in images taken after injection of cet.Hum.scFv-IRDye800CW and cetuximab-IRDye800CW. The tumor-to-background ratio was significantly higher in A-431 tumor-bearing mice receiving cet.Hum.scFv-IRDye800CW than those receiving cetuximab-IRDye800CW. According to data from recombinant antibody fragments of other antibodies [42], cet.Hum.scFv-IRDye800CW had higher blood clearance rate than cetuximab-IRDye800CW, producing more condensed signal at the tumor site. Studies comparing full-length antibodies and antibody fragments in fluorescence-guided surgery are rare. El-Sayed and colleagues have reported that full-length anti-HER3 IgG and (scFv)-Fc fusion protein are more potent than scFv, scFv-CH3, diabody, and Fab fragments in imaging of HNSCC xenografts [42]. When compared to cet.Hum.scFv-IRDye800CW, cetuximab-IRDye800CW needed a longer time to produce its maximum signal intensity, likely due to its larger size which slows tumor penetration. Antibody fragments (e.g., Fab, minibodies, diabodies, and scFv) have been used as targeting agents in fluorescence-guided surgery for a variety of cancers, including head and neck cancer, colon cancer, prostate cancer, pancreatic cancer, and breast cancer [30, 32, 47–50]. Schoonooghe and colleagues have reported that even smaller antibody fragments (referred to as nanobodies and heavy-chain variable region of antibodies) can be used as potential imaging agents [51]. U-87 MG cancer cells express low levels of EGFR [39]. As expected, none of IRDye800CW-conjugated molecules was able to detect U-87 tumor xenografts in an efficient manner. Injection of fluorescent molecules resulted in dispersed signals throughout the mouse body. 24 h after injection, cet.Hum.scFv-IRDye800CW resulted in faint signal at the sites of U-87 MG tumors, which gradually disappeared within the next 48 h. Xu and colleagues have reported that anti-EGFR Fab did not accumulate in low EGFR expression cells (M14 cells) but was able to recognize EGFR-overexpressing A-431 cells [52].

Cetuximab-IRDye800CW was also unable to detect U-87 MG tumors at all. These results confirm that cet.Hum.scFv-IRDye800CW has a higher tumor penetration ability than RDye800CW-conjugated cetuximab. Considering the specificity of cet.Hum.scFv-IRDye800CW toward EGFR-overexpressing cells and also the less tissue penetration ability of infrared wavelengths (compared to X-rays, gamma rays, and so on), it will be useful only for imaging of superficial human cancers that overexpress EGFR. Squamous cell carcinoma of head and neck (SCCHN) is a group of cancer that usually overexpresses EGFR molecules. These cancers are also superficial enough to be imaged using infrared dye-conjugated antibodies like cet.Hum.scFv-IRDye800CW.

Previous studies have shown that fluorophore conjugation can lead to affinity reduction [53–55]. Fluorescence quantum yield parameter for antibody-conjugated fluorophores has been reported to be lower than that of free fluorophores [53]. In this study, we labeled both cet.Hum.scFv and cetuximab, with NIR dye IRDye800CW, at 1.983 and 2.128 dyes/antibody, respectively. *K*_d_ values of both cet.Hum.scFv and cetuximab, unexpectedly, increased after IRDye800CW conjugation. Although it is usual, *K*_d_ rising following dye conjugation has already been reported. Bernhard and colleagues have reported that *K*_d_ values of (scFv)2, scFv-Fc, and an IgG increased after IRDye800CW conjugation but did not point out how this shifting in *K*_d_ value occurs.

**Figure 8:**
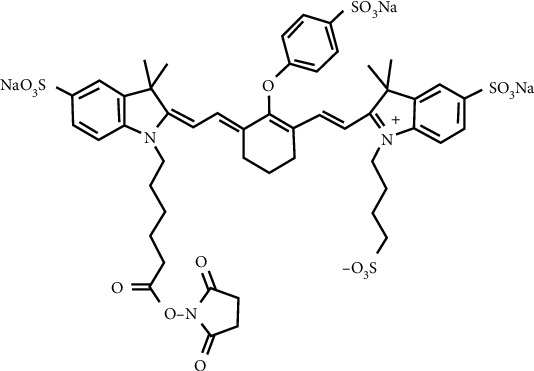
Molecular structure of IRDye800CW NHS ester (https://www.licor.com/bio/reagents/irdye-800cw-nhs-ester).

Complementary determining regions (CDRs) in antibody molecules are responsible for antigen recognition. There are a number of amino acids in CDR loops of both cet.Hum.scFv and cetuximab which have primary or secondary amino group(s) at their side chains. Based on the information provided by LI-COR (https://www.licor.com/bio/reagents/irdye-800cw-nhs-ester), IRDye800CW binds to proteins via their side chain primary and secondary amino groups. The dye (Figure 8) has a number of potential sites that are prone to form hydrogen bonds with side chains of some amino acids (e.g., tyrosine, arginine, and lysine). The higher the number of hydrogen bonds between the antibody and antigen, the higher the *K*_d_ value may be obtained.

Tumor imaging using IRDy800 CW has several advantages over noninfrared fluorescent dye (e.g., FITC) as well as some infrared dyes like 5-aminolevulinic acid (5-ALA). IRDy800CW has near-infrared spectrum absorption and emission wavelengths (774-794 nm). So, autofluorescence observed with 5-ALA and visible light fluorescent dyes does not occur tangibly when working with IRDy800 CW [56]. Altogether, the result of this study indicates that molecular probe consisting of an anti-EGFR cet.Hum.scFv and IRDye800CW is able to recognize EGFR-overexpressing tumor cells in an efficient manner and can be a good candidate for further studies in the hope of developing a new molecular probe for tumor imaging.

## Data Availability

No dataset was generated during this study.
